# Ovarian reserve of women with and without BRCA pathogenic variants: A systematic review and meta-analysis

**DOI:** 10.1016/j.breast.2021.09.006

**Published:** 2021-09-27

**Authors:** Maria Luisa Gasparri, Rosa Di Micco, Veronica Zuber, Katayoun Taghavi, Giampaolo Bianchini, Serena Bellaminutti, Francesco Meani, Rossella Graffeo, Massimo Candiani, Michael D. Mueller, Andrea Papadia, Oreste D. Gentilini

**Affiliations:** a Department of Gynecology and Obstetrics, Ospedale Regionale di Lugano EOC, via tesserete 46, 6900 Lugano, Switzerland; b Faculty of Biomedical Sciences, Università della Svizzera Italiana, via Giuseppe Buffi 13, 6900, Lugano, Switzerland; c Breast Surgical Unit, San Raffaele University Hospital, Milan, Italy; d Department of Clinical Medicine and Surgery, University of Naples Federico II, Naples, Italy; e Institute of Social and Preventive Medicine, University of Bern, Bern, Switzerland; f Department of Medical Oncology, San Raffaele University Hospital, Milan, Italy; g Institute of Oncology of Southern Switzerland (IOSI) and Breast Unit of Southern Switzerland (CSSI), Bellinzona, Switzerland; h Department of Gynecology and Obstetrics, San Raffaele University Hospital, Milan, Italy; i Department of Obstetrics and Gynecology, University Hospital of Bern and University of Bern, Bern, Switzerland

**Keywords:** AMH, Breast cancer, Ovarian reserve, BRCA1m, BRCA2m, BRCA pathogenic variants

## Abstract

**Introduction:**

Preliminary clinical evidence suggests a detrimental effect of pathogenic variants of BRCA1 and 2 genes on fertility outcome. This meta-analysis evaluates whether women carrying BRCA mutations (BRCAm) have decreased ovarian reserve, in terms of Anti-Muellerian Hormone (AMH), compared to women without BRCAm (wild-type).

**Material and methods:**

Systematic searches of PubMed, Medline, Scopus, Embase, Science Direct and the Cochrane Library from inception until July 2020 were conducted. All studies comparing AMH level in fertile age women, with and without BRCA pathogenic variants were considered. Sub-analyses were performed according to age, presence of breast cancer, and type of mutation.

**Results:**

Among 64 studies, 10 series were included. For the entire cohort, a trend of reduced AMH level were found between BRCAm carriers and women without pathogenic variants. BRCAm carriers aged 41-years or younger had lower AMH levels compared to 41-years or younger wild type women (OR: 0.73 [95%CI-1.12;-0.35]; p = 0.0002). This finding was confirmed for BRCA1m carriers (OR: 1 [95%CI-1.96;-0.05]; p = 0.004) whereas no difference was observed between BRCA2m carriers and wild type women. The same analysis on breast cancer patients with and without BRCAm achieved the same results.

**Conclusion:**

Young BRCA1m carriers seem to have lower AMH level compared with wild type women and therefore a potential decreased ovarian reserve.

## Highlights

• BRCAm carriers have similar AMH levels to wild-type women.
• In women under 42 years old, AMH level is statistically lower in BRCA1m.
• Fertility preservation counselling should be offered once BRCA pathogenic variant is diagnosed.

## Graphical abstract

**Figure:**
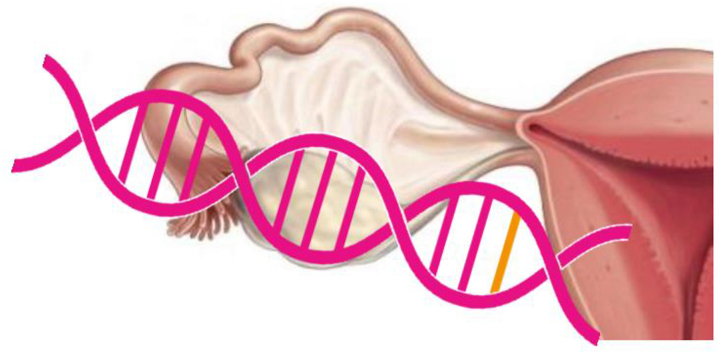

## 1 Introduction

T he lifetime risk of ovarian and breast cancer (BC) in women with pathogenic variants in BRCA1 and BRCA2 gene is 72% and 69%, 44% and 17%, respectively [1]. NCCN guidelines recommend a risk-reducing removal of ovaries and fallopian tubes between ages 35 and 40 and upon completion of child bearing for BRCA1 mutation (BRCA1m) carriers. Delaying risk-reducing removal of ovaries and fallopian tubes until age 40–45 is reasonable for women carrying BRCA2 mutation (BRCA2m) because the average age of ovarian cancer onset is 8–10 years later than in BRCA1m [2]. The implications of BRCA pathogenic variants on women's reproductive health are still uncertain. Therefore, no clear data can be specifically provided to patients facing this fight against biological timing.

BRCA1 and BRCA2 genes play a key role in DNA double-strand breaks (DBSs), chromosomal stability, apoptosis and cell cycle [3]. Therefore, a deficiency in these genes may result in meiotic errors and predispose to apoptosis [4]. In vitro experiments showed that BRCA pathogenic variants could lead to oocyte apoptosis and premature follicular depletion [5]. BRCA 1-mutant mice showed lower number of primordial follicle and a reduced response to ovarian stimulation [6]. In breast cancer patients undergoing ovarian stimulation for fertility preservation, patients carrying BRCA pathogenic variants achieved poorer response to ovarian stimulation compared to non-mutated breast cancer patients [7,8]. Some authors reported comparable ovarian reserve and responses to ovarian stimulation in BRCAm carriers with and without breast cancer, compared with women with BRCA negative cancer and cancer free controls [9,10]. On the contrary, recent studies on BRCAm carriers have shown less ovarian reserve and a higher rate of earlier onset of menopause on average than women without the pathogenic variant [11,12].

Anti-Mullerian Hormone (AMH) is a biomarker of ovarian reserve. It reflects ovarian aging and is one of the most relevant indicators of women's fertility potential [13]. AMH plays a role in the primary follicle depletion rate and its values appear to correspond well with antral follicle counts and fertility potential [14,15]. It also correlates with the number of oocytes retrieved following ovarian stimulation for fertility treatment [16]. AMH decreases with age and predicts age of menopause [17,18].

The predictive value of the ovarian reserve and whether lower AMH in BRCAm carriers places breast cancer patients at a higher risk for chemotherapy-induced ovarian failure were recently investigated. Presence of a deleterious BRCA germline mutation did not affect AMH level in breast cancer patients undergoing chemotherapy and endocrine therapy [19].

The aim of the meta-analysis was to compare AMH levels as a marker of ovarian reserve between BRCAm carriers and women without mutations (wild type).

## 2 Material and methods

### 2.1 Data identification and selection

This systematic review and meta-analysis was performed according to the Preferred Reporting Items for Systematic reviews and Meta-Analyses (PRISMA) statement. On July 2020, the systematic literature search was performed. All eligible studies were included without restriction on publication year and/or language. The studies were identified using the electronic databases PubMed, Medline, Scopus, Embase, Science Direct, and the Cochrane Library adopting the search terms “AMH” or “anti-mullerian hormone” and “BRCA” “BRCA1” “BRCA2” and “ovarian reserve”. All original articles comparing the AMH level of BRCAm carriers and wild type were included. The study groups were the following: BRCAm breast cancer women and not affected BRCAm carriers. The control groups consisted of breast cancer and cancer-free women tested for BRCA and resulted negative. All women were in fertile age, assessed by regular menses. Only women tested for BRCA have been considered for the analysis. In all eligible studies, as well as in our analysis, the outcome was AMH as the marker of ovarian reserve. Reference lists of already published reviews and original reports were also analyzed in order to identify potential studies. Review articles, case reports, studies on animal models and protocols, were excluded. For studies not reporting relevant data, such as standard deviation and/or mean, the first/last/corresponding authors of these studies were questioned in order to provide additional information. The authors who confidentially provided these data were acknowledged in the specific section and their studies were included in the analysis. The studies from authors unable to provide this missing information were excluded from the analysis.

### 2.2 Data extraction and outcomes

For each study included in the meta-analysis, the following data were recorded: first author's information and publication year, type of patients (breast cancer patients undergoing fertility preservation/cancer-free women tested for BRCA undergoing surveillance program), number of BRCAm women/total, study groups available for comparison, type of the study, age of patients (range), major matching criteria/adjusted variables. The primary outcome was the ovarian reserve, defined by the level of AMH in ng/mL. It was reported in mean+/-standard deviation for each study and control group. Sub-analyses have been performed based on age and presence of breast cancer.

When the series reported the AMH level of women carrying BRCA1 and 2 pathogenic variants as a unique mean value, no sub-analyses for BRCA1m and BRCA2m were performed. When BRCA1m and 2 data were also presented separately, a sub-analysis on BRCA1m and BRCA2m was performed. When studies reported data only on BRCA1m or BRCA2m, these data were considered only for the respective sub-analyses and not for the cumulative BRCA1/2 m forest plot.

The following comparisons were considered: 1. BRCAm 1 and 2 carriers with or without breast cancer (study group) versus wild type women with or without breast cancer (control group); 2. BRCAm carriers with or without breast cancer under 42 years old (study group) versus not mutation carriers (wild type) with or without breast cancer under 42 years old (control group); 3. BRCA1m carriers with and without breast cancer (study group) versus wild type women with and without cancer (control group); 4. BRCA1m carriers with and without breast cancer under 42 years old (study group) versus wild type women with and without cancer under 42 years old (control group); 5. BRCA2m carriers with and without breast cancer (study group) versus wild type women with and without cancer (control group); 6. BRCA2m carriers with and without breast cancer under 42 years old (study group) versus wild type women with and without cancer under 42 years old (control group); 7. BRCA1m breast cancer patients undergoing fertility preservation under 42 years old (study group) versus wild type breast cancer patients undergoing fertility preservation under 42 years old (control group); 8. BRCA2m breast cancer patients undergoing fertility preservation under 42 years old (study group) versus wild type breast cancer patients undergoing fertility preservation under 42 years old (control group).

### 2.3 Statistical analysis

A χ2 test for heterogeneity among proportions was performed to verify the presence of statistical heterogeneity between studies. The pooled odds ratio (OR) was calculated using a fixed- or random-effects model, according to the most suitable model to the sampling frame, and not on the basis of the heterogeneity. Under the fixed-effects model, we assumed one true effect size that underpinned all the studies included in that analysis, and that all differences in observed effects were due to sampling error. Under the random-effects model we allowed that the true effect size might differ from study to study and a more intensive variant of intervention was used. Briefly, under fixed-effects model it was reasonable to assume that studies were similar enough and that there was a common effect. Furthermore, the fixed-effects model was used only when the number of studies included in the analysis was less than five. Graphical representation of each study and pooled analysis was displayed by forest plots. The weight that each study provides in the meta-analysis was graphically reported as a square of different size. Confidence intervals (CIs) for each study were symbolized as a horizontal line passing through the square. The pooled OR was represented as a lozenge in the forest plot and its size corresponded to the 95% CI of the OR. A p value ≤ 0.05 was considered significant. The data extracted from the studies included in the meta-analysis were analyzed using Review Manager (RevMan), Version 5.3, Copenhagen: The Nordic Cochrane Centre, The Cochrane Collaboration, 2014.

## 3 Results

Overall, 64 studies were identified through the literature search. Of these, 27 studies were removed as duplicates and 37 were screened. Of these, 30 were excluded after title and abstract evaluation because 9 were reviews, 2 were case reports, 1 was a protocol proposal, 6 studies had different aims, 3 studies were in vitro molecular analyses or carried out on animals, two articles were case reports, 1 study did not report the AMH levels and in another three papers the AMH levels were only reported as median (instead of mean) or with mean without standard deviation [[20], [21], [22]], and 1 article focused on endometriosis. One study apparently fulfilling inclusion criteria, categorized women as low risk due to a negative family history for breast and/or ovarian cancer, without a confirmation BRCA test, therefore it was excluded [23]. At the end of the screening, ten studies resulted eligible and were included in the meta-analysis [5,6,10,[24], [25], [26], [27], [28], [29], [30]]. The group populations of the ten selected studies, considered as upper limit 40 [[24], [25], [26]], 42 [6], and 45 years old [5,[27], [28], [29], [30]]. Age related sub-analyses were performed from the higher age (45 years) to the lower until a statistical significance was detected. A statistical significance was detected at the cut-off of 42 years of age.

**Fig. 1:**
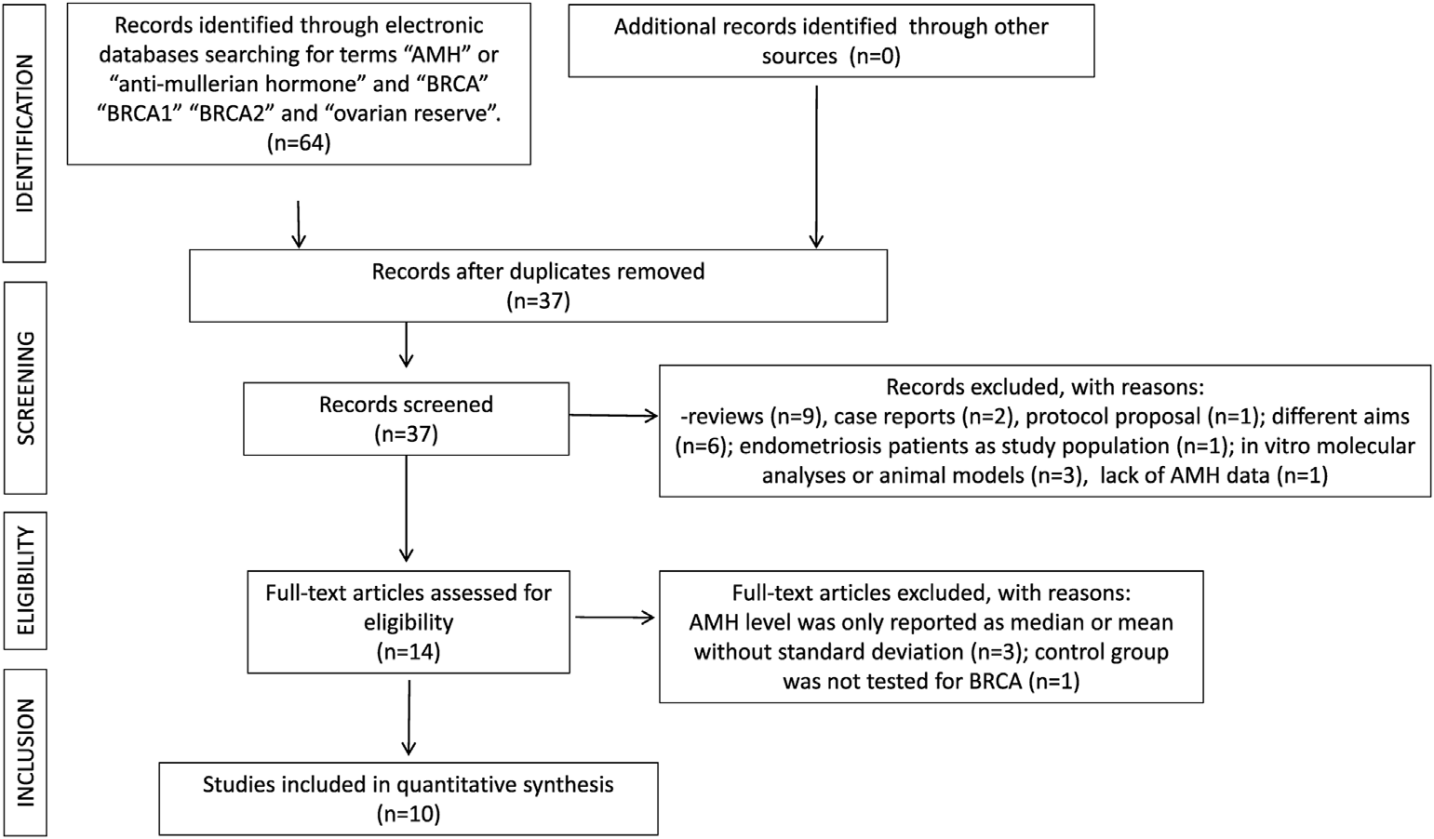
PRISMA flow diagram on the Meta-analysis process.

Fig. 1 represents the PRISMA flow diagram of the identification, screening, eligibility, and inclusion process described above. Overall, 1644 women tested for BRCAm were included in the analysis. Of those tested for BRCA, 830/1644 (50.5%) were BRCA/12m carriers. The characteristics of these ten studies are reported in Table 1. Based on the Cochrane risk-of-bias assessment, some studies exhibited significant bias mostly in terms of the personnel blinding process because of the nature of the interventions (Table 2).

**Table 1:** Characteristics of the included studies.

**Authors, year**	**Setting**	**Program**	**BRCAm/Tot∗**	**Study groups available for comparison**	**Design of the study**	**Age**	**Restriction or matching for major confounding factors/adjusted variables**		
Titus S, 2013	BC	ART (FP)	24/84	15 BRCAlm BC	Prospective	18–42	Age, ovarian dysfunction		
				9 BRCA2m BC					
				BRCAm 1 + 2					
				60 WT					
Wang ET, 2014	Cancer-free	S	89/143	62 BRCA1m	Cross-sectional study	18–45	Age, BMI, smoke, HC		
				27 BRCA2m					
				BRCAm 1 + 2					
				54 WT					
Phillips KA, 2016	Cancer-free	S	319/535	216 WT	Prospective	25–45	Age, BMI, smoke, HC		
				172 BRCA1m					
				147 BRCA2m					
Giordano S, 2016	Cancer-free	S	68/124	68 BRCA1m	Retrospective	18–45	Age, BMI, HC, smoke, PCOS		
				56 WT					
Johnson L, 2017	Cancer-free	S	105/131	55 BRCA1	Prospective	18–45	Age, BMI, HC, smoke		
				50 BRCA2					
				26 WT					
Ben-Aharon I, 2018	Cancer-free	S	33/48	33 BRCAm 1 + 2	Prospective	24–40	Age		
				15 WT					
Porcu E, 2019	BC	ART (FP)	22/46	11 BRCA1 BC	Prospective	18–40	Age, BMI		
				11 BRCA2 BC					
				24 WT BC					
Gunnala V, 2019	Cancer- free and BC"	ART (FP)	49/102	38 BRCAm 1 + 2 (BC)	Retrospective	Up to 40		Age, BMI	
				53 WT (BC)					
				*BRCAm sub-groups:*					
				31 BRCAm 1 (BC + cancer-free) 18 BRCAm 2 (BC + cancer-free)					
Grynberg, 2019	BC	ART (FP)	52/329	52 BRCAm 1 + 2	Retrospective	18–40		Age, BMI	
				277 WT					
Ponce 2020	Cancer- free	S	69/135	32 BRCA1	Retrospective	18–45		Age, PCOS, smoke, BMI, HC	
				37 BRCA2					
				66 WT					

BC: breast cancer; S: surveillance; ART: assisted reproductive technology; FP: fertility preservation; WT: wild type; HC: hormonal contraceptive use.

∗tot. = BRCAm + wild type (confirmed by BRCA test).

# The cancer-free BRCAm carriers (n = 19) were not considered for the comparison with the cancer-free group without mutation (cancer-free control group).

(n = 600), since the cancer-free control group was not tested for BRCA and was defined only as low risk.

**Table 2:** Bias assessment.

**Type of Bias**	**Selection Bias**	**Performance Bias**	**Detection Bias**	**Attrition Bias**
**Study**				
Titus S, 2013	low	low	low	low
Wang ET, 2014	intermediate	low	intermediate	intermediate
Phillips KA, 2016	intermediate	low	low	low
Giordano S, 2016	high	low	intermediate	low
Johnson L, 2017	high	low	low	low
Ben-Aharon I, 2018	intermediate	low	low	low
Porcu E, 2019	low	low	low	low
Gunnala V, 2019	intermediate	low	intermediate	high
Grynberg, 2019	intermediate	low	intermediate	low
Ponce 2020	high	low	intermediate	low

### 3.1 AMH in BRCA1/2m carriers with and without breast cancer versus wild type women

**Fig. 2:**
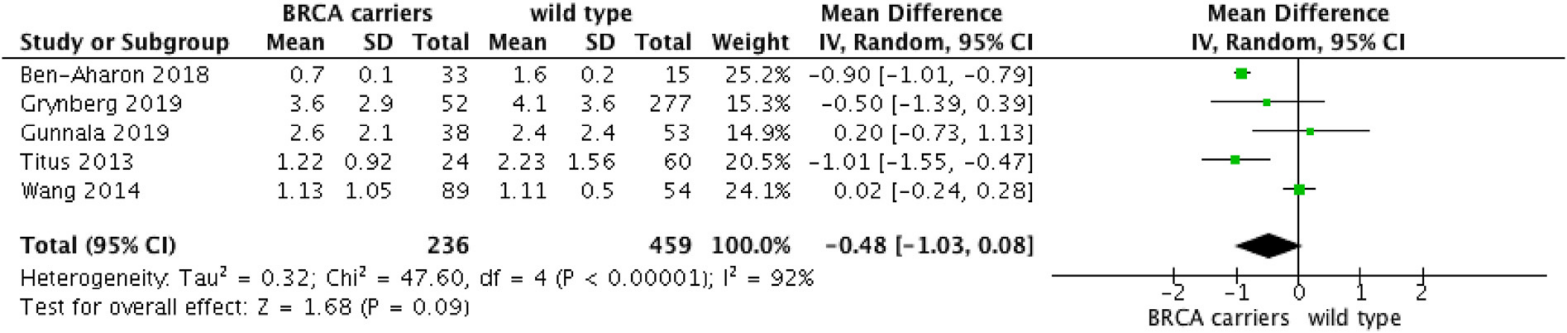
AMH in BRCAm 1/2 breast cancer and cancer-free women versus wild-type non cancer women.

**Fig. 3:**
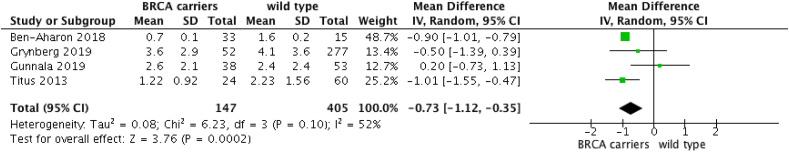
AMH in BRCAm 1/2 breast cancer and cancer-free women versus wild-type non cancer women in under 42y

When comparing AMH of women carrying BRCA1-2 pathogenic variants with and without breast cancer to wild type women with and without breast cancer, five studies were included [5,6,10,24,26]. The analysis revealed that BRCAm carriers have similar AMH levels to wild-type women (OR: −0.48 [95%CI -1.03; 0.08]; p = 0.09), despite a trend of reduced AMH levels in wild-type women (Fig. 2). However, at an exploratory analysis including only women aged under 42 years, AMH level is statistically lower in BRCAm women (OR: −0.73 [95%CI -1.12; −0.35]; p = 0.0002) (Fig. 3).

#### 3.1.1 ✓ BRCA1m

**Fig. 4:**
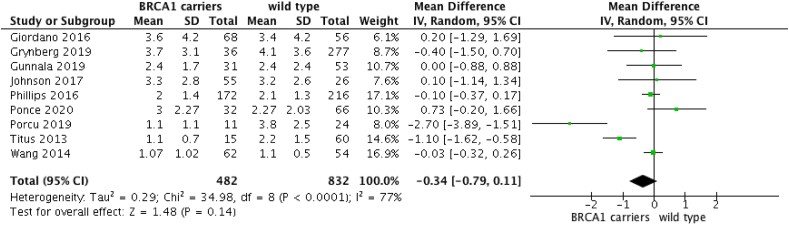
AMH in BRCA1m breast cancer and cancer-free women versus wild-type non cancer women.

**Fig. 5:**
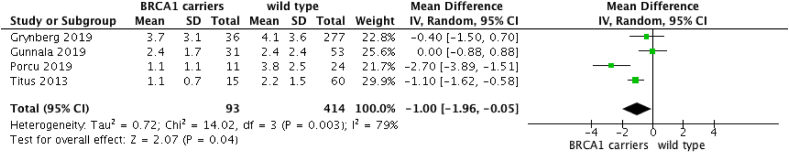
AMH in BRCA1m breast cancer and cancer-free women versus wild-type non cancer women in under 42y

When comparing AMH of BRCA1m breast cancer patients to wild type breast cancer patients, nine studies were included [5,6,10,[25], [26], [27], [28], [29], [30]]. The analysis revealed that BRCA1m carriers have similar AMH levels to wild type women (OR: −0.34 [95%CI -0.79; 0.11]; p = 0.14) (Fig. 4). However, when women under 42 years are selected, AMH level is statistically lower in BRCA1m women (OR: −1 [95%CI -0.05; −0.35]; p = 0.04) (Fig. 5).

#### 3.1.2 ✓ BRCA2m

**Fig. 6:**
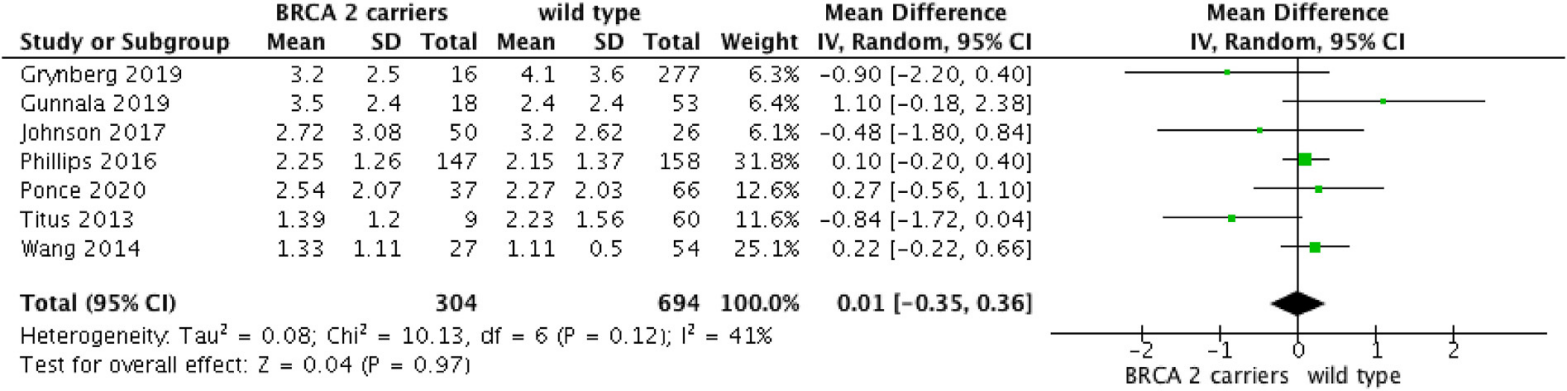
AMH in BRCA2m breast cancer and cancer-free women versus wild-type non cancer women.

**Fig. 7:**
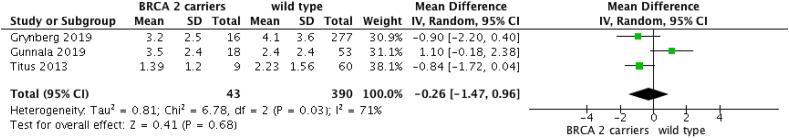
AMH in BRCA2m breast cancer and cancer-free women versus wild-type non cancer women in under 42.

When comparing AMH of BRCA2m breast cancer patients to wild-type breast cancer patients, seven studies were included [5,6,10,26,27,29,30]. The analysis revealed that BRCA2m carriers have similar AMH levels compared to wild type women (OR: 0.01 [95%CI -0.35; 0.36]; p = 0.97) (Fig. 6). No differences were found when a sub-analysis of only women under 42 years was performed (OR: −0.26 [95%CI -1.47; 0.96]; p = 0.68) (Fig. 7).

### 3.2 AMH in breast cancer patients undergoing fertility preservation in under 42y

**Fig. 8:**
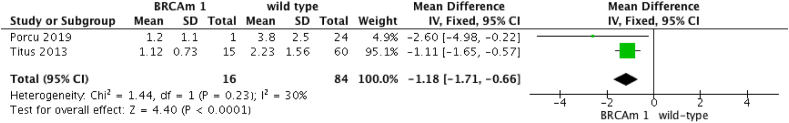
AMH in breast cancer patients undergoing fertility preservation in under 42y: BRCA1m versus wild-type.

**Fig. 9:**
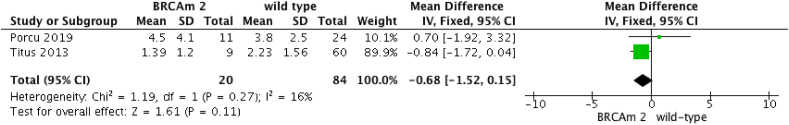
AMH in breast cancer patients undergoing fertility preservation in under 42y: BRCA2m versus wild-type.

When comparing AMH of BRCA1m and BRCA2m breast cancer patients under age 42 to wild type breast cancer patients, two studies were included [6,25]. The AMH level of BRCA1m patients was statistically lower compared to wild type breast cancer patients (OR: −1.18 [95%CI -1.71; −0.66]; p < 0.0001) (Fig. 8. No differences between AMH level of BRCA2m breast cancer and wild type breast cancer patients were found (OR: −0.68 [95%CI -1.52; 0.15]; p = 0.11) (Fig. 9).

## 4 Discussion

The results of this meta-analysis carried out on 1644 women overall showed a non-significant trend of reduction in AMH levels comparing patients with or without BRCAm. However, an exploratory analysis including only patients of younger age demonstrated a decrease in AMH levels among BRCAm. In particular, when BRCAm 1 and 2 carriers were analyzed separately, only women under 42 carrying a BRCA1 pathogenic variant showed a significantly lower AMH level compared to wild type women. This finding was also apparent when breast cancer patients with and without BRCA pathogenic variants undergoing fertility preservation were analyzed separately to breast cancer-free women. When the comparisons included also older women, the significance was missed. These data may be interpreted by the AMH decrease with increasing aging, making it difficult to get a significant p-value in older women.

Young women carrying the BRCA1 or BRCA2 pathogenic variants have challenging decisions regarding their reproductive life and childbearing future and require careful counseling on this topic [31,32]. Once the BRCA1m is detected, NCCN guidelines clearly recommend risk-reducing bilateral salpingo-oophorectomy between ages 35 and 40 and upon completion of childbearing [2]. The early onset of menopause in these patients is currently a topic of interest and strategies to reduce the symptoms are currently promoted [33]. However, many women are nulliparous when they receive this diagnosis and there is no specific recommendation for routine evaluation of fertility potential and ovarian reserve in female BRCAm carriers. Currently, indications to ART for BRCAm carriers, are the same as for wild type women.

BRCA genes are tumor suppressor genes, acting to ensure the integrity of the genome through the repair of homologous recombination (HR). On one side, the deficient HR repair causes premature depletion of the primordial ovarian pool [6]. Results from transgenic mice on the impairment of heterologous recombination repair were confirmed in BRCA1m carriers with accelerated loss of ovarian follicular reserve and accumulation of DNA double-strand breaks in oocytes. On the other side, BRCA genes maintain the chromosome telomere integrity and telomeres, which shorten after each cycle of DNA replication, being associated with ovarian aging and reproductive senescence [[34], [35], [36]]. Considering that factors affecting cell cycle division and DNA repair may also affect reproductive life span, it might be reasonable that defects in BRCA genes may affect reproduction.

Although BRCAm carriers are more likely to have a poor response to ovarian stimulation [7], Grynberg at al. have recently shown that BRCA1/2 pathogenic variants do not seem to affect the capacity of oocytes to mature in vitro in breast cancer patients candidate for fertility preservation [26]. When assessing the ovarian reserve in vivo, the marker that best reflects the gradual decline in reproductive capacity with increasing age is the AMH [37].

In our meta-analysis, the presence of the BRCA1 pathogenic variant did not affect AMH level significantly in the analysis without age restriction, but the trend towards lower AMH level was close to statistical significance. The significance was achieved for BRCA1m when only women under 42 years were considered. AMH level in BRCA2m was not influenced by age, also when the age was lowered at 42 and 40 years old (data not shown).

Wang et al. suggest that the difference between BRCA1m and BRCA2m carriers may be attributable to a differential effect on ovarian reserve as the epidemiology of ovarian cancer differs between the two pathogenic variants as well [5]. Firstly, BRCA1 pathogenic variants carry a higher lifetime risk of ovarian cancer occurring over a decade earlier than BRCA2-associated ovarian cancers [1,38]. This difference in age penetrance is mirrored by in vitro studies of age-related decline in BRCA1 gene expression in human oocytes from reproductive-age women [6]; however, this was not noted for BRCA2 gene expression, speculating that the decline may occur in later years. Secondly, BRCA1 and BRCA2 have distinct molecular functions in the DNA repair pathway and BRCA2 plays a more limited role in homologous recombination repair, thus anticipating its minor impact on oocyte depletion. There are limited data regarding oocyte quality of BRCAm carriers, and future investigations may be focused on this topic.

We acknowledge some inherent limits to this study. As is typical for meta-analyses, biases may derive from the inclusion criteria of each study. When comparing women with and without BRCA pathogenic variants undergoing fertility preservation, all women with a history of non-breast cancer and/or ovarian surgery, gonadotoxic therapy before presentation, ovarian dysfunction (e.g. PCOS), were excluded in order to avoid interference with AMH level. However, interference with AMH level may persist in some cases. Further limit may be related to the different methodology adopted in the studies for the AMH testing. Until recently, AMH has been measured with an enzyme-linked immunosorbent assay (ELISA), which is a manual technique characterized by large analytical variation; however, automated assays have recently been introduced for measuring AMH.

Giordano et al. evaluated only BRCA1m carriers and found reduced AMH levels in >35 year-old women only [28], however in order to avoid bias when comparing these data with the other series, we included the mean value of the entire cohort that was confidentially provided from the authors we contacted. Other authors considered patients from 18 or 25y to 40 or 45y together, however, all the studies were matched/adjusted for age. Other confounders were also considered in the studies (e.g. BMI, PCOS, smoke, use of hormonal therapy), however it is reasonable to assume that internal biases in the study and control group may persist. As for instance, Gunnala et al. found a large difference in mean age between the BRCA carriers and BRCA non carriers, despite the groups being adjusted for age [10].

Furthermore, case and control women underwent BRCA testing because of personal or familiar history of cancer and results were negative, according to genetic counseling. However, we cannot exclude that other kinds of unexplored genetic alterations may interfere with the reduction in ovarian reserve to a greater extent than BRCA genes. In BRCAm breast cancer patients, determining the difference with wild type women in terms of age at menopause, and pregnancy rate might be biased by the gonadotoxicity of cancer treatments, similarly the risk reducing strategies might affect the cancer free BRCAm women. Therefore, these data are still missing and not in the scope of our study.

## 5 Conclusion

Young BRCA1m carriers seem to have lower AMH levels compared with wild type women.

It is unclear whether the lower AMH levels we observed translate into a reduced ovarian reserve and pregnancy rate. Additionally, if the awareness of the BRCA pathogenic variants unconsciously leads to an anticipated median age of conception is still to be investigated. Given the impossibility to design randomized clinical trials with this aim, this type of study represents the best available evidence that can be achieved. If further studies will confirm this hypothesis, once the pathogenic variant is diagnosed, physicians might consider to initiate an infertility work-up and address these women to a fertility preservation program with a faster pace. Additionally, If the decreased ovarian reserve in BRCAm1 will be confirmed in future trials, the possible increased risk of gonadotoxicity in breast cancer patients carrying the BRCA1 pathogenic variant should be another issue to consider when counseling these patients.

## Declaration of competing interest

The authors have nothing to declare. They have no conflict of interest.
